# Smurf2 regulates hematopoietic stem cell self-renewal and aging

**DOI:** 10.1111/acel.12195

**Published:** 2014-02-04

**Authors:** Charusheila Ramkumar, Yahui Kong, Sally E Trabucco, Rachel M Gerstein, Hong Zhang

**Affiliations:** 1 Department of Cell and Developmental Biology, University of Massachusetts Medical SchoolWorcester, MA, 01655, USA; 2 Microbiology and Physiological Systems, University of Massachusetts Medical SchoolWorcester, MA, 01655, USA

**Keywords:** aging, hematopoietic stem cells, self-renewal, senescence, Smurf2

## Abstract

The age-dependent decline in the self-renewal capacity of stem cells plays a critical role in aging, but the precise mechanisms underlying this decline are not well understood. By limiting proliferative capacity, senescence is thought to play an important role in age-dependent decline of stem cell self-renewal, although direct evidence supporting this hypothesis is largely lacking. We have previously identified the E3 ubiquitin ligase Smurf2 as a critical regulator of senescence. In this study, we found that mice deficient in *Smurf2* had an expanded hematopoietic stem cell (HSC) compartment in bone marrow under normal homeostatic conditions, and this expansion was associated with enhanced proliferation and reduced quiescence of HSCs. Surprisingly, increased cycling and reduced quiescence of HSCs in Smurf2-deficient mice did not lead to premature exhaustion of stem cells. Instead, HSCs in aged Smurf2-deficient mice had a significantly better repopulating capacity than aged wild-type HSCs, suggesting that decline in HSC function with age is Smurf2 dependent. Furthermore, Smurf2-deficient HSCs exhibited elevated long-term self-renewal capacity and diminished exhaustion in serial transplantation. As we found that the expression of *Smurf2* was increased with age and in response to regenerative stress during serial transplantation, our findings suggest that Smurf2 plays an important role in regulating HSC self-renewal and aging.

## Introduction

Aging is a complex process of gradual deterioration of physiological functions that involves virtually all cells and tissues. Tissue-specific stem cells, which are capable of self-renewal to preserve stem cell pools as well as differentiation into a variety of effector cells, play a central role in the normal homeostatic maintenance and regenerative repair of tissues throughout the lifetime of an organism. With advancing age, the self-renewal capacity of stem cells invariably declines, eventually leading to the accumulation of unrepaired, damaged tissues in old organisms (Rando, 2006; Sharpless & DePinho, 2007; Rossi *et al*., 2008). Aging of the hematopoietic system is driven by many intrinsic and extrinsic factors that adversely affect the production and functions of blood cells (Van Zant & Liang, 2003; Chambers & Goodell, 2007; Rossi *et al*., 2008). Increasing evidence suggests that attenuated hematopoietic stem cell (HSC) function plays an important role in hematopoietic aging, including anemia, decreased immune function, and increased propensity for myeloid malignancies. The ability of HSCs to repopulate and reconstitute lethally irradiated recipients is limited in serial transplantation, reflecting a finite potential of HSC self-renewal under regenerative stress (Ogden & Mickliem, 1976; Harrison & Astle, 1982). In aged mice, HSCs undergo distinct changes: They exhibit decreased repopulating ability, even though the number of phenotypic HSCs based on staining of cell surface markers increases with age in some laboratory mouse strains. Further, aged HSCs show a differentiation preference toward myeloid lineages at the expense of lymphoid differentiation (Morrison *et al*., 1996; Sudo *et al*., 2000; Liang *et al*., 2005; Rossi *et al*., 2005; Chambers *et al*., 2007). In humans, aged HSCs also show increased frequency and a bias toward myeloid potential (Pang *et al*., 2011).

The molecular mechanisms responsible for age-dependent decline in HSC self-renewal are complex and many factors have been implicated (Van Zant & Liang, 2003; Chambers & Goodell, 2007; Rossi *et al*., 2008). Senescence, a state of irreversible growth arrest that most cells enter at the end of their replicative lifespan, has been proposed to contribute to aging by reducing the renewal capacity of stem cells (Sharpless & DePinho, 2004; Campisi, 2005). Although direct evidence supporting the senescence theory of aging is lacking, several senescence regulators including p16^INK4a^ (p16) and p53 have been found to play important roles in aging. The expression of *p16* increases with age in many human and rodent tissues (Krishnamurthy *et al*., 2004; Ressler *et al*., 2006). Age-associated increase in *p16* in mice coincides with a decline in the renewal capacity of stem cells in bone marrow, brain, and pancreas (Janzen *et al*., 2006; Krishnamurthy *et al*., 2006; Molofsky *et al*., 2006), although *p16* up-regulation in aged HSCs has been challenged (Attema *et al*., 2009). HSCs in old mice lacking *p16* have increased regenerative potential, suggesting that p16 plays a critical role in limiting HSC self-renewal (Janzen *et al*., 2006). In *p53*-null mice, the number of HSCs increases and they perform better in competitive repopulation, suggesting an enhanced self-renewal capacity (TeKippe *et al*., 2003; Liu *et al*., 2009). Moreover, mice with one wild-type allele and one mutant allele of *p53* that lacks the N-terminal transactivation domain maintain cancer protection, but age prematurely including impairment of HSCs (Tyner *et al*., 2002; Maier *et al*., 2004; Dumble *et al*., 2007). These studies support an emerging link between senescence regulation and aging and show the potential importance of senescence regulation in the aging of HSCs.

We have recently shown that the E3 ubiquitin ligase Smurf2 plays a critical role in regulating the senescence response in human and mouse cells (Zhang & Cohen, 2004; Kong *et al*., 2011; Ramkumar *et al*., 2012). Adventitious expression of *Smurf2* is sufficient to induce senescence in early passage cells (Zhang & Cohen, 2004; Ramkumar *et al*., 2012), while deficiency in *Smurf2* expression impairs the senescence response in culture and *in vivo* (Kong *et al*., 2011; Ramkumar *et al*., 2012). In this study, we found that *Smurf2* deficiency led to increased proliferation and an expanded HSC compartment in bone marrow. Surprisingly, increased proliferation did not lead to early HSC exhaustion. Instead, Smurf2-deficient HSCs showed better repopulating ability and multilineage potential than wild-type cells with advancing age or under regenerative stress, suggesting a functional role of Smurf2 in the regulation of HSC self-renewal and aging.

## Results

### Increased expression of *Smurf2* in mouse bone marrow during aging

**Figure 1:**
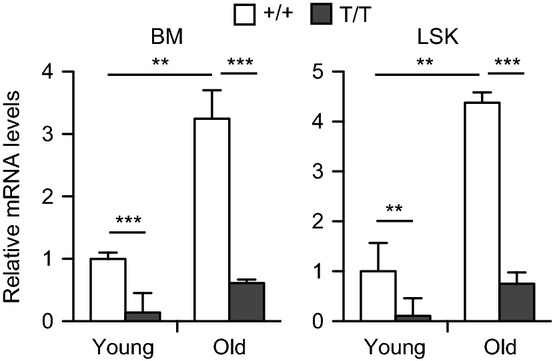
Increased *Smurf2* expression in aged mice. Quantitative RT–PCR analysis of *Smurf2* expression in bone marrow (BM) and sorted LSK (Lin^−^Sca1^+^c-kit^++^) cells of young (2-month) and old (24-month) wild-type (+/+) and *Smurf2*^*T/T*^ (T/T) mice. Relative expression in young wild-type cells was set to be 1 after normalization with β-actin. Error bars are SD of three independent experiments. Student’s *t*-test is used for statistical analysis. **P < 0.01, ***P < 0.001.

We have shown previously that Smurf2 is an important regulator of senescence (Zhang & Cohen, 2004; Kong *et al*., 2011; Ramkumar *et al*., 2012). To investigate whether Smurf2 plays a role in aging, we first examined the expression of *Smurf2* in mouse bone marrow (BM) and the LSK (Lin^−^Sca-1^+^c-kit^++^; Lin^−^: negative for lineage markers B220, CD3, CD11b, CD19, Gr-1, and Ter-119) population that is enriched for HSCs (Ikuta & Weissman, 1992; Okada *et al*., 1992; Osawa *et al*., 1996). We found that *Smurf2* expression was increased in total BM and LSK cells of aged (24-month) C57BL/6 mice compared with young (2-month) mice (Fig. 1).

We have generated a Smurf2-deficient mouse model (*Smurf2*^*T/T*^, T for the trapped allele), in which a gene-trapping cassette is inserted into intron 1 of *Smurf2* to disrupt its normal splicing (Ramkumar *et al*., 2012). The expression of *Smurf2* was significantly reduced in total BM and LSK cells of Smurf2-deficient mice compared with wild-type (WT) mice (Fig. 1). Because of the hypomorphic nature of the trapped *Smurf2* allele, there were residual normal splicing and *Smurf2* expression in BM, LSK cells (Fig. 1), common lymphoid progenitors, multipotent progenitors, and HSCs (Fig. S1A) of Smurf2-deficient mice, similar to what we have found previously in other tissues (Ramkumar *et al*., 2012). Reduced Smurf2 expression in this mouse model allowed us to investigate whether Smurf2 has a functional role in HSC aging.

### Expansion of long-term HSCs in Smurf2-deficient mice

**Figure 2:**
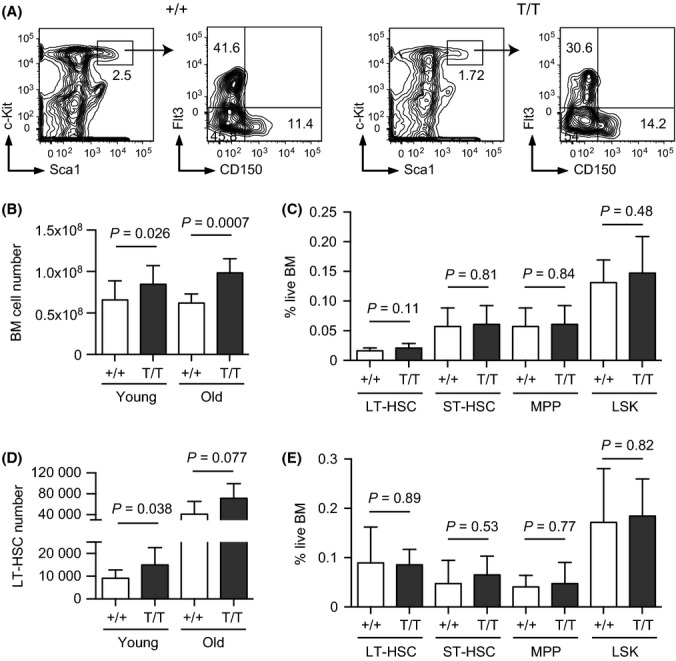
Increased bone marrow (BM) cellularity and expanded long-term hematopoietic stem cell (LT-HSC) population in Smurf2-deficient mice. (A) Representative flow cytometry analysis of HSCs in BM of 2-month-old wild-type (+/+) and *Smurf2*^*T/T*^ (T/T) mice. Live lineage-negative (Lin-) cells are gated and displayed for staining of Sca1 and c-Kit (left panels). LSK (Lin^−^Sca1^+^c-kit^++^) population is gated and displayed for staining of CD150 and Flt3 (right panels). LT-HSCs are defined as Lin^−^Sca1^+^c-kit^++^CD150^+^Flt3^−^, while Lin^−^Sca1^+^c-kit^++^CD150^−^Flt3^−^ and Lin^−^Sca1^+^c-kit^++^CD150^−^Flt3^+^ are used to enumerate short-term HSCs (ST-HSCs) and multipotent progenitors (MPPs), respectively. (B) Average live BM cell numbers in young (2-month, *N* = 16) and old (24- to 30-month, *N* = 7) mice. (C) Frequencies of LT-HSC, ST-HSCs, MPP, and LSK cells as percent of live BM cells in young (2-month-old, *N* = 11) mice. (D) Average LT-HSC numbers in young (2-month, *N* = 11) and old (24- to 30-month, *N* = 6) mice. (E) Frequencies of LT-HSC, ST-HSCs, MPP, and LSK cells as percent of live BM cells in old (24- to 30-month, *N* = 6) mice. Error bars are SD and Student’s *t*-test is used for statistical analysis.

To investigate the role of Smurf2 in HSC aging, we used flow cytometry to enumerate BM populations enriched for long-term HSCs (LT-HSCs) (Lin^−^Sca1^+^c-kit^++^CD150^+^Flt3^−^), short-term HSCs (ST-HSCs: Lin^−^Sca1^+^c-kit^++^CD150^−^Flt3^−^), and multipotent progenitors (MPPs: Lin^−^Sca1^+^c-kit^++^CD150^−^Flt3^+^) (Adolfsson *et al*., 2001; Wilson *et al*., 2007; Papathanasiou *et al*., 2009) (Fig. 2A). We noticed a statistically significant increase (1.28-fold, *P* = 0.026) in the total live BM cells collected from long bones of hind and forelegs of 2-month-old Smurf2-deficient mice compared with age-matched WT mice (Fig. 2B), whereas gross body weights were not significantly different between WT and Smurf2-deficient mice (Fig. S1B). Although no significant difference in the frequencies of LT-HSCs, ST-HSCs, MPPs, or LSK population was found between young WT and Smurf2-deficient mice (Fig. 2C), the total number of LT-HSCs in young Smurf2-deficient mice was significantly increased (1.64-fold, *P* = 0.038) compared with WT mice (Fig. 2D). ST-HSCs, MPPs, or LSK cells were also increased (1.30–1.45-fold) in young Smurf2-deficient mice, although the increases were not statistically significant (Fig. S2A).

In aged (24- to 30-month) mice, we also found an increase in BM cellularity in Smurf2-deficient mice (1.59-fold, *P* = 0.0007) compared with WT mice (Fig. 2B). Similar to what we found in young mice, the frequencies of LT-HSCs, ST-HSCs, MPPs, and LSK population were not significantly different between aged WT and Smurf2-deficient mice (Fig. 2E). Although the average numbers of LT-HSCs, ST-HSCs, MPPs, and LSK populations increased (1.73–2.21-fold) in aged Smurf2-deficient mice, there were great variations among individual aged mice and the increases were not significantly different from those in WT mice (Fig. 2 and Fig. S2B).

Complete blood count analyses of peripheral blood showed no significant difference between age-matched WT and Smurf2-deficient mice at either young (2 months) or old (18–20 months) age in various lineages, including white blood cells, red blood cells, lymphocytes, granulocytes, monocytes, and platelets (Fig. S3). These results suggest that differentiation and commitment of HSCs to various lineages are not affected by Smurf2 deficiency.

### Enhanced cell proliferation of LT-HSCs in Smurf2-deficient mice

**Figure 3:**
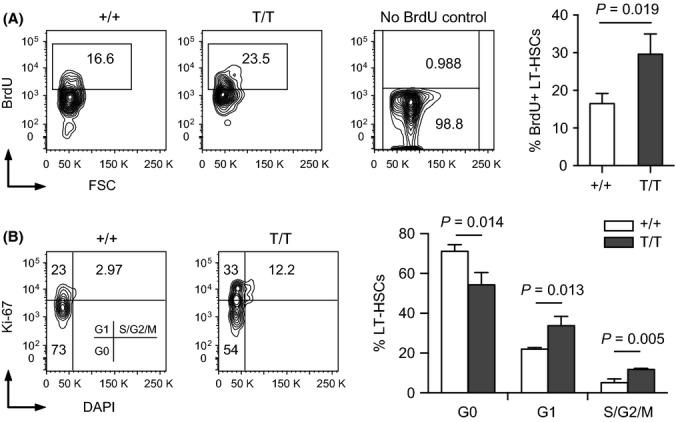
Increased proliferation and decreased quiescence of Smurf2-deficient hematopoietic stem cells (HSCs). (A) Representative BrdU incorporation analysis of HSCs in wild-type (+/+) and *Smurf2*^*T/T*^ (T/T) mice. Long-term HSCs (Lin^−^Sca1^+^c-kit^++^CD150^+^) are gated and displayed for BrdU staining. BM from mice without BrdU injection was stained for BrdU as a control. Average of three independent experiments is shown at right. (B) Representative cell cycle analysis of HSCs in wild-type (+/+) and *Smurf2*^*T/T*^ (T/T) mice. Long-term HSCs (Lin^−^Sca1^+^c-kit^++^CD150^+^) are gated and displayed for staining of Ki-67 and DAPI. Average of three independent experiments is shown at right. Error bars are SD and Student’s *t*-test is used in statistical analysis.

The observed increases in BM cells and LT-HSCs in Smurf2-deficient mice suggest an enhanced cell proliferation of these cells. Supporting this notion, we found that the fraction of BM cells in the S phase of cell cycle was increased in 2-month-old Smurf2-deficient mice compared with WT mice (Fig. S4A; 11.9 ± 0.7% in Smurf2-deficient vs. 8.1 ± 0.4% in WT, *P* = 0.0012). To further investigate whether cell proliferation is enhanced in Smurf2-deficient LT-HSCs and progenitor populations, we gave young mice 4 injections of bromodeoxyuridine (BrdU) in a span of 24 h to label LT-HSCs (Passegue *et al*., 2005). BrdU was incorporated in a significant portion of LT-HSCs (Lin^−^Sca1^+^c-kit^++^CD150^+^) using this labeling method (Fig. 3). We found that BrdU incorporation was significantly increased in Smurf2-deficient LT-HSCs (1.8-fold, *P* = 0.019) compared with WT LT-HSCs (Fig. 3). Increased BrdU incorporation was also observed in Smurf2-deficient LSK and ST-HSC/MPP (Lin^−^Sca1^+^c-kit^++^CD150^−^) populations compared with WT cells (Fig. S4B).

As most LT-HSCs in adult mice remain quiescent (Cheshier *et al*., 1999), we combined Ki-67 staining with cell cycle analysis to determine the frequencies of LT-HSCs that are quiescent or cycling (Fig. 3). Smurf2-deficient LT-HSCs (Lin^−^Sca1^+^c-kit^++^CD150^+^) exhibited decreased frequency in the G0 phase (54.2% in Smurf2-deficient vs. 71.2% in WT; *P* = 0.014), and increased frequencies in the G1 (33.8% in Smurf2-deficient vs. 22.1% in WT; *P* = 0.013) and S-G2/M phases (11.8% in Smurf2-deficient vs. 5.1% in WT; *P* = 0.005) (Fig. 3). Similarly, Smurf2-deficient LSK and ST-HSC/MPP (Lin^−^Sca1^+^c-kit^++^CD150^−^) populations also showed significantly increased cycling and decreased quiescence (Fig. S4C). We did not observe LT-HSCs in sub-G1 phase, suggesting that there is no apoptosis in these cells. Collectively, these data indicate that cell proliferation is enhanced in Smurf2-deficient LT-HSCs.

### Enhanced repopulation ability of HSCs in aged Smurf2-deficient mice

**Figure 4:**
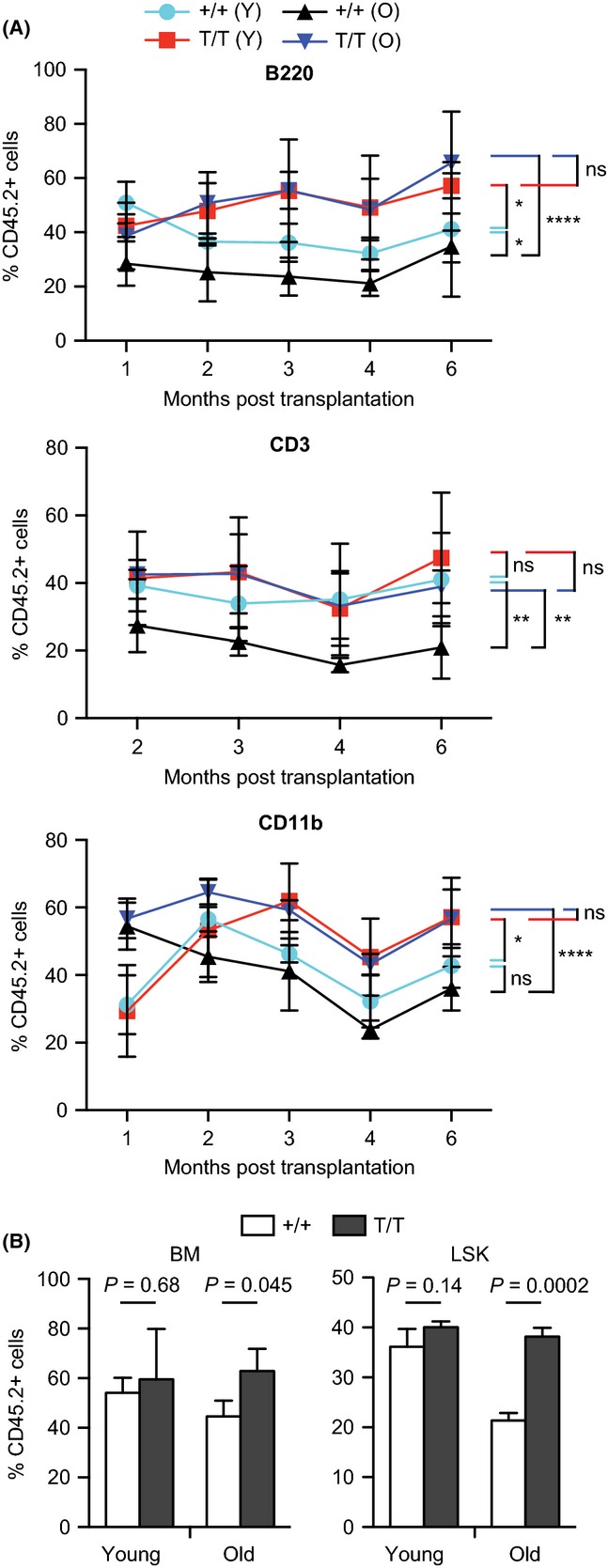
Hematopoietic stem cells (HSCs) in aged Smurf2-deficient mice exhibit enhanced repopulating ability. Donor bone marrow (BM) from wild-type (+/+) or *Smurf2*^*T/T*^ (T/T) mice (CD45.2^+^) mixed at 1:1 ratio with competitor BM of 2-month-old wild-type (CD45.1^+^/CD45.2^+^) mice are injected into lethally irradiated recipient mice (CD45.1^+^). Contribution of donors is determined by CD45 staining. Both young (2-month) and old (24-month) donors were used. (A) Donor (CD45.2^+^) contributions to B (B220^+^), T (CD3^+^), and myeloid (CD11b^+^) lineages in peripheral blood of recipient mice (*N* = 4) at indicated time post-BM transplantation. Two-way anova is used for statistical analysis. *P < 0.05, **P < 0.01, ****P < 0.0001, ns: not significant. (B) Donor contribution to BM and LSK (Lin^−^Sca1^+^c-kit^++^) cells in recipient mice (*N* = 3) at 6 months post-BM transplantation. Error bars are SD. Student’s *t*-test is used in statistical analysis.

As quiescence has been postulated to prevent premature stem cell exhaustion during aging (Orford & Scadden, 2008), our observation of decreased frequency of quiescent LT-HSCs in Smurf2-deficient mice suggests that Smurf2-deficient HSCs may experience premature exhaustion. To determine the self-renewal ability and multilineage potential of HSCs, we used competitive repopulation (Harrison, 1980), in which donor BM cells of either WT or Smurf2-deficient mice (CD45.2^+^) were mixed at a 1:1 ratio with competitor BM cells of 2-month-old CD45.1^+^/CD45.2^+^ WT mice and injected into lethally irradiated congenic recipient mice (CD45.1^+^). Contribution of donor, competitor, and recipient in peripheral blood and BM of reconstituted recipients was determined by flow cytometry at different time post-transplantation. As we have found previously that ~30% of Smurf2-deficient mice develop tumor spontaneously after 12 month of age, and ~70% of tumors are B-cell lymphomas in spleen (Ramkumar *et al*., 2012), only mice that were tumor free were used in this study. We selected aged mice that were physically fit without any sign of stress or illness and did not have visible tumors upon necropsy. Furthermore, we used flow cytometry to characterize spleen from these aged mice to make sure that there was no lymphoma as described (Ramkumar *et al*., 2013). Smurf2-deficient HSCs of young (2-month) mice were more efficient in repopulating B and myeloid cells than young WT HSCs and as efficient as WT HSCs in repopulating T cells in the peripheral blood of the reconstituted recipient mice (Fig. 4). Interestingly, Smurf2-deficient HSCs of aged (24-month) animals showed significantly better repopulation ability than the age-matched WT HSCs (Fig. 4), despite the decreased quiescence and increased cycling of Smurf2-deficient LT-HSCs. In fact, old Smurf2-deficient HSCs had similar repopulating ability as the young HSCs (Fig. 4). Furthermore, analysis of BM and LSK population 6 months after transplantation showed increased contribution from aged Smurf2-deficient donor compared with WT donor (Fig. 4). Collectively, these results indicate that HSCs in aged Smurf2-deficient mice have better repopulation ability and suggest that Smurf2 deficiency protects HSCs from early exhaustion during aging.

### Smurf2-deficient LT-HSCs exhibit better long-term self-renewal capacity under regenerative stress

To investigate the long-term self-renewal and multilineage potential of HSCs, we used BM serial transplantation, in which LT-HSCs in donor BM are able to reconstitute lethally irradiated recipients in successive but limited transplantations, reflecting the finite potential of HSC self-renewal (Ogden & Mickliem, 1976; Harrison & Astle, 1982). As the frequency of LT-HSCs in Smurf2-deficient mice was not significantly different from WT mice, we used the same number of BM cells from 2-month-old WT and Smurf2-deficient mice in transplantation. We used either female or male donors in two independent experiments. During the 1st, 2nd, or 3rd transplantation, 100% of recipient mice receiving donor BM from WT or Smurf2-deficient mice survived. Recipient mice from the 3rd transplantation were followed for a year and none of them died during observation. Staining of BM cells in the recipient mice during these successive transplantations revealed that >75% of the LT-HSC population had been reconstituted by the donor (Fig. S5A). Three months after transplantation, complete blood count analysis of peripheral blood of recipient mice showed that various blood cell lineages were successfully reconstituted by both the WT and Smurf2-deficient donors (Fig. S5B).

**Figure 5:**
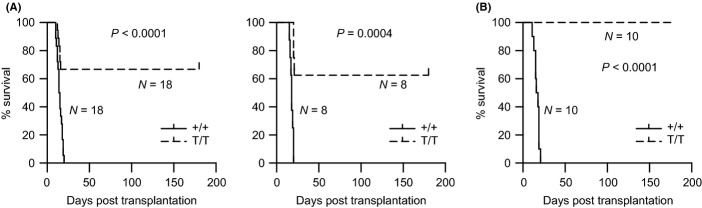
Increased long-term self-renewal capacity and multilineage potential in Smurf2-deficient hematopoietic stem cells (HSCs). Bone marrow (BM) cells of wild-type (+/+) and *Smurf2*^*T/T*^ (T/T) mice are used as donors in serial BM transplantation. (A) Kaplan–Meier survival curves of recipient mice receiving BM from 2-month-old donors in the 4th transplantation cycle. Two independent serial transplantation experiments using male (left panel) or female (right panel) donors are shown. (B) Kaplan–Meier survival curves of recipient mice receiving BM from 24-month-old donors in the 4th transplantation cycle. The log-rank test is used for statistical analysis.

During the 4th transplantation, all recipients receiving BM from WT mice died within 3 weeks post-transplantation. In contrast, recipients receiving BM from Smurf2-deficient mice showed significantly better survival (Fig. 5; 63–67% survival, *P* < 0.001), suggesting that Smurf2-deficient HSCs have an enhanced long-term self-renewal capacity. None of these recipient mice died during 1 year of observation. We carried out a 5th transplantation with Smurf2-deficient donors and found that 60% of the recipient mice survived for 1 year post-transplantation without signs of illness or stress (Fig. S6).

Similarly, we carried out BM serial transplantation using aged mice as donors. All recipients receiving donor BM from either aged WT or Smurf2-deficient mice survived during the first 3 cycles of transplantation. Complete blood count analysis of recipient peripheral blood 3 months after transplantation showed that various blood cell lineages were successfully reconstituted by both the WT and Smurf2-deficient donors (Fig. S5C). During the 4th transplantation, all recipients receiving BM from WT mice died within 3 weeks post-transplantation, while 100% recipients receiving BM from Smurf2-deficient mice survived more than 6 months post-transplantation (Fig. 5; *P* < 0.0001). Collectively, these results indicate that Smurf2-deficient LT-HSCs have a better capacity in the long-term self-renewal and multilineage potential than the WT LT-HSCs. Furthermore, this stem cell-intrinsic functional enhancement is maintained in aged Smurf2-deficient mice.

### Smurf2 regulates *p16* expression during aging and under regenerative stress

**Figure 6:**
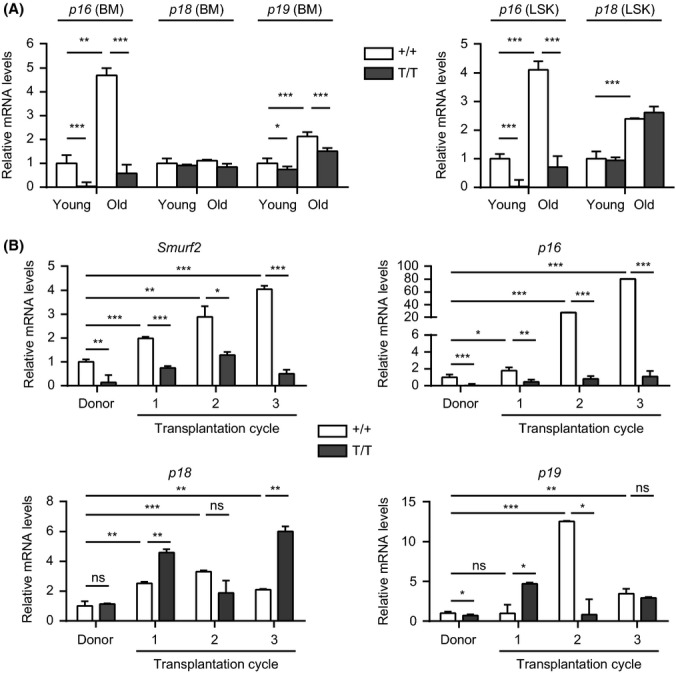
Age or regenerative stress-induced *p16* expression is attenuated in Smurf2-deficient mice. (A) Quantitative RT–PCR analysis of gene expression in bone marrow (BM) and sorted LSK (Lin^−^Sca1^+^c-kit^++^) cells of young (2-month) and old (24-month) wild-type (+/+) and *Smurf2*^*T/T*^ (T/T) mice. Relative expression in young wild-type cells was set to be 1 after normalization with β-actin. Only *P* < 0.05 are indicated in pairwise comparison. (B) Quantitative RT–PCR analysis of gene expression in BM of donors and successive recipients in serial transplantation. Relative expression in wild-type donors is set to be 1 after normalization with β-actin. Error bars are calculated from standard deviations of three independent experiments. Student’s *t*-test is used for statistical analysis. *P < 0.05, **P < 0.01, ***P < 0.001, ns: not significant.

Enhanced long-term self-renewal of Smurf2-deficient HSCs despite increased cycling and decreased quiescence in these cells prompted us to examine the expression of cyclin-dependent kinase inhibitors, as several of them have been found to regulate HSC self-renewal. HSCs lacking *p18*^*INK4c*^ (*p18*) have increased self-renewal ability accompanied by increased cycling (Yuan *et al*., 2004), and *p16* has been shown to limit HSC self-renewal during aging (Janzen *et al*., 2006). We found that *p18* expression was not changed, while the expression of *p16* and *p19*^*Arf*^ (*p19*), which are located in the same *INK4a* locus, was increased in BM of aged (24-month) WT C57BL/6 mice compared with young (2-month) mice (Fig. 6), consistent with a previous report (Krishnamurthy *et al*., 2004). Interestingly, the expression of *p16* and *p18* was increased in LSK cells with age, whereas *p19* expression was undetectable in LSK cells (Fig. 6). As our recent studies show that Smurf2 regulates the expression of *p16* during senescence (Kong *et al*., 2011), we found that *p16* expression was significantly suppressed in BM and LSK cells in Smurf2-deficient mice (Fig. 6). In contrast, the expression of *p19* showed a slight decrease and *p18* was largely unchanged in Smurf2-deficient cells (Fig. 6).

We then analyzed the expression of these genes as well as *Smurf2* in BM of reconstituted recipients during BM serial transplantation. We found that *Smurf2* expression was increased during successive transplantation in mice receiving WT donor BM, concomitant with an increase in the expression of *p16*, *p18,* or *p19* (Fig. 6). While the increase in *p18* and *p19* expression was not consistently affected by Smurf2 deficiency, the increase in p16 expression was greatly attenuated in mice receiving the Smurf2-deficient donor BM (Fig. 6), suggesting a possible link between p16 and Smurf2-mediated regulation of HSC self-renewal and aging.

## Discussion

An important characteristic of HSCs in adult animals is their relative quiescence (Cheshier *et al*., 1999; Mahmud *et al*., 2001). It is thought that quiescence prevents premature stem cell exhaustion during aging (Orford & Scadden, 2008). Many quiescence regulators have been found to control stem cell aging. For example, cyclin-dependent kinase inhibitor 1A (CDKN1A or p21) is required to maintain quiescence of HSCs. In the absence of *p21*, increased cell cycling leads to premature exhaustion and impaired self-renewal of HSCs (Cheng *et al*., 2000). Conversely, deletion of transcriptional factor *ELF4*, a negative regulator of quiescence, leads to increased quiescence and reduced exhaustion of HSCs (Lacorazza *et al*., 2006). In this study, we found that Smurf2 deficiency led to enhanced cell proliferation in BM. HSCs in Smurf2-deficient mice exhibited increased cell cycling and decreased quiescence. Surprisingly, increased proliferation of HSCs in Smurf2-deficient mice did not lead to premature stem cell exhaustion. Instead, Smurf2-deficient HSCs showed increased long-term self-renewal and multilineage repopulating capacity under regenerative stress in serial transplantation compared with WT HSCs. Furthermore, HSCs in aged Smurf2-deficient mice displayed better repopulating capability than HSCs in aged WT mice. Taken together, our results indicate that Smurf2 deficiency mitigates age-dependent decline in HSC self-renewal and function despite increased HSC cycling.

We found that *Smurf2* expression was increased in BM and LSK population enriched for HSCs with advancing age or in response to regenerative stress in serial transplantation, concomitant with increases in the expression of *p16*, *p19,* and *p18*. HSCs lacking *p18* have increased long-term self-renewal capacity, even though these cells show increased proliferation (Yuan *et al*., 2004). Our finding that *p18* expression was unchanged by *Smurf2* deficiency suggests that *p18* is unlikely responsible for Smurf2-regulated decline in HSC self-renewal with age. It is documented that *p16* expression increases with age in many human and rodent tissues, including BM and HSCs (Krishnamurthy *et al*., 2004; Janzen *et al*., 2006; Ressler *et al*., 2006), although age-dependent increase in *p16* in HSCs has been challenged (Attema *et al*., 2009). In old mice, *p16*^*−/−*^ HSCs perform much better than their WT counterparts in serial transplantation, suggesting that age-dependent increase in *p16* expression limits HSC self-renewal (Janzen *et al*., 2006). We have recently found that Smurf2 regulates p16 expression during senescence (Kong *et al*., 2011). In Smurf2-deficient mice, age or regenerative stress-induced *p16* expression was significantly attenuated, suggesting a possible link between HSC self-renewal and *p16* expression regulated by Smurf2. Supporting this notion, we found that Smurf2-deficient HSCs of aged mice substantially outperformed aged WT HSCs in competitive repopulation and serial transplantation. In fact, old Smurf2-deficient HSCs performed as well as young HSCs, a phenotype similarly observed in old *p16*^*−/−*^ HSCs (Janzen *et al*., 2006). However, Smurf2-deficient HSCs in young mice behaved differently from young *p16*^*−/−*^ HSCs, which are more readily depleted in serial transplantation than WT HSCs (Janzen *et al*., 2006). In contrast, HSCs in young Smurf2-deficient mice also outperformed WT HSCs in serial transplantation and competitive repopulation. It is possible that Smurf2 limits HSC self-renewal in a p16-dependent manner only in aged mice, or Smurf2 deficiency protects age-dependent decline in HSC self-renewal independently of p16. Additional studies are needed to address the precise role of p16 in Smurf2-mediated regulation of HSC self-renewal and aging.

In complex organisms such as mammals, many somatic tissues such as bone marrow are capable of renewal, repair, and even regeneration. Renewable tissues allow the replacement of damaged cells, offering a clear advantage over postmitotic tissues. It is important for stem cells to maintain lifetime self-renewal and proliferation. In this study, we found that *Smurf2* expression was increased with advancing age in BM and LSK cells. More importantly, HSCs in old Smurf2-deficient mice maintain their self-renewal capacity at the level similar to young HSCs, suggesting that functional decline in HSC self-renewal during aging is Smurf2 dependent. We have previously shown that Smurf2 is an important regulator of senescence (Zhang & Cohen, 2004; Kong *et al*., 2011; Ramkumar *et al*., 2012). Increased expression of *Smurf2* is sufficient to induce senescence (Zhang & Cohen, 2004; Ramkumar *et al*., 2012), while loss of *Smurf2* expression impairs the senescence response in culture and *in vivo* (Kong *et al*., 2011; Ramkumar *et al*., 2012). Senescence is proposed to contribute to aging by depleting the renewal capacity of tissues and/or by interfering with tissue homeostasis and functions (Sharpless & DePinho, 2004; Campisi, 2005). Although cells with characteristics of senescence have been detected to accumulate with age *in vivo*, their functional contribution to aging is still not completely clear (Sharpless & DePinho, 2004; Campisi, 2005). Our study provides evidence for Smurf2 in regulating HSC self-renewal and aging, further strengthening the emerging link between senescence and stem cell aging.

## Experimental procedures

### Smurf2-deficient mice

Smurf2-deficient mice as described previously (Ramkumar *et al*., 2012) had been backcrossed to C57BL/6 (CD45.2^+^) for more than 10 generations. All mouse studies were carried out according to guidelines approved by the Institutional Animal Care and Use Committee of University of Massachusetts Medical School.

### Complete blood count

Peripheral blood was bled from mouse tail veins into EDTA-coated tubes, and complete blood count analysis was performed using the Heska CBC-Diff Veterinary Hematology System (Heska, Loveland, CO, USA).

### Flow cytometry

Bone marrow cells were harvested from long leg bones and resuspended in staining medium consisting of biotin-, flavin-, and phenol red-deficient RPMI 1640 medium (Invitrogen, Carlsbad, CA, USA) supplemented with 2% FBS (Hyclone, Logan, UT, USA), 10 mM HEPES (pH 7.2), 1 mM EDTA, and 0.02% sodium azide. After filtering through 70-μm nylon mesh, BM cells were incubated with anti-CD16/32 antibody (BioXCell, West Lebanon, NH, USA) for 10 min on ice to block Fc receptors and then incubated with primary antibodies for 20 min. The lineage cocktail contained biotin-conjugated antibodies to B220 (RA3-6B2), CD3e (145-2C11), CD11b (M1/70), CD19 (1D3), Ly-6G (RB6-8C5), and Ter-119 (TER-119). Additional antibodies for HSC analysis were Ly-6A/E(Sca1)-FITC or Ly-6A/E(Sca1)-PerCp-Cy5.5 (D7), CD117(c-Kit)-PE-Cy7 or CD117(c-Kit)-APC (2B8), CD135(Flt3)-PE (A2F10), and CD150-APC (mShad150). All antibodies were purchased from eBioscience (San Diego, CA, USA). Cells stained with biotin-labeled antibodies were incubated with streptavidin-eFluor 450 (eBioscience) for 15 min on ice and washed three times with staining medium. After the final wash, cells were resuspended in staining medium with 1 μg mL^-1^ propidium iodide to exclude dead cells. Flow cytometry analysis was performed on a 5-laser, 18-detector LSR II FACS (BD Biosciences, San Jose, CA, USA), and data were analyzed using FlowJo software (Treestar, Ashland, OR, USA).

### Cell cycle and cell proliferation analyses

Mice were injected with 1 mg BrdU intraperitoneally every 6 h for 24 h as described previously (Passegue *et al*., 2005). BM cells were stained for HSCs as described above, fixed and permeabilized using the BrdU flow kit (BD Biosciences). These cells were then stained with anti-BrdU-FITC or anti-Ki-67-PerCp-Cy5.5 (Sola15) antibody for 20 min. Cells stained for Ki-67 were further incubated with DAPI for 30 min at room temperature as described (Yang *et al*., 2008). Stained cells were analyzed by flow cytometry as described above.

### Serial transplantation and competitive repopulation

Wild-type or Smurf2-deficient mice (CD45.2^+^) were used as donors, and congenic CD45.1^+^ mice (The Jackson Laboratory, Bar Harbor, ME) were used as recipients. Eight- to ten-week-old recipient mice were lethally irradiated (10 Gy) using a Cs^137^ irradiator 24 h before transplantation and were treated with antibiotics (0.5 mg mL^-1^ neomycin and 100 U mL^-1^ polymyxin-B) in drinking water 24 h prior to exposure to radiation until 1 month after transplantation. For serial transplantation, 5 × 10^6^ donor BM cells were injected retro-orbitally into 8- to 10-week-old lethally irradiated recipient mice. The transplantation cycle was repeated every 2 months.

For competitive repopulation, 2-month-old WT CD45.1^+^/CD45.2^+^ mice were used as competitors. 1 × 10^6^ donor BM cells were mixed with 1 × 10^6^ competitor BM cells and injected into 8- to 10-week-old lethally irradiated recipient mice. The relative contributions from the donors, competitors, or recipients in peripheral blood and BM of reconstituted recipients were analyzed in flow cytometry using antibodies to CD45.2-FITC (104) and CD45.1-PE-Cy7 (A20). Multilineage reconstitution in peripheral blood was analyzed using antibodies to B220-APC (RA3-6B2), CD3e-biotin (145-2C11), and CD11b-APC-Cy7 (M1/70, BD Biosciences). Antibodies were purchased from eBioscience unless specified.

### Quantitative RT–PCR

Total RNA was isolated from freshly collected BM or FACS-sorted LSK cells using TRIzol (Invitrogen) and reverse-transcribed using Superscript II reverse transcriptase (Invitrogen). Real-time PCR was carried out on MyiQ iCycler using SYBR Green PCR kit (Bio-Rad, Hercules, CA). The following primers were used: Smurf2 (5′-ATGAAGTCATTCCCCAGCAC-3′; 5′-AACCGTGCTCGTCTCTCTTC-3′), p16 (5′-CGAACTCTTTCGGTCGTACCC-3′; 5′-CGAATCTGCACCGTAGTTGAG-3′), p18 (5′-GGGGACCTAGAGCAACTTACT-3′; 5′-AAATTGGGATTAGCACCTCTGAG-3′), p19 (5′- GCTCTGGCTTTCGTGAACATG-3′; 5′-TCGAATCTGCACCGTAGTTGAG-3′), and β-actin (5′-GCTCTTTTCCAGCCTTCCTT-3′, 5′-GTGCTAGGAGCCAGAGCAGT-3′).

### Statistical analysis

Data were presented as mean ± SD. Two-tailed and unpaired Student’s *t*-test was used for pairwise comparisons. Two-way anova was used for multiple comparisons. Kaplan–Meier survival curves were plotted using GraphPad Prism 5.0, and statistical significance was analyzed using the log-rank test. *P* < 0.05 was considered as statistically significant.

## Supporting Information

**Fig. S1** Smurf2 expression in sorted HSCs, MPPs, CLPs, and body weights of wild-type and Smurf2-deficient mice.

**Fig. S2** Average numbers of progenitor populations in bone marrows of wild-type and Smurf2-deficient mice at young and old ages.

**Fig. S3** Complete blood count analysis of peripheral blood in wild-type and Smurf2-deficient mice at young and old ages.

**Fig. S4** Cell proliferation of bone marrow cells and progenitor populations in wild-type and Smurf2-deficient mice.

**Fig. S5** Donor contributions to bone marrow and complete blood count analysis of peripheral blood in recipient mice during serial transplantation.

**Fig. S6** Survival of recipient mice receiving bone marrow from 2-month-old Smurf2-deficient donors in the 5th transplantation cycle.
