# Supplementary material for: Smurf2 regulates hematopoietic stem cell self-renewal and aging
Source: Aging Cell. 2014 Feb 4;13(3):478–86. doi: 10.1111/acel.12195 (PMC4032599; doi:10.1111/acel.12195)
Supplement: Supplementary file 7 [file acel0013-0478-sd7.doc]

**Supplemental Figure Legends**

**Fig. S1** (A) Quantitative RT-PCR analysis of Smurf2 expression in sorted hematopoietic stem cells (HSC: Lin^-^CD127^-^Sca1^+^c-kit^++^Flt3^-^), multi-potent progenitors (MPP: Lin^-^CD127^-^Sca1^+^c-kit^++^Flt3^+^) and common lymphoid progenitors (CLP: Lin^-^CD127^+^AA4.1^+^Sca1^+^c-kit^+^Flt3^+^) of 2-month old wild-type (+/+) and *Smurf2^T/T^* (T/T) mice. Relative expression in wild-type cells was set to be 1 after normalization with β-actin. Error bars are SD of 3 independent experiments. (B) Average body weight of 2-month old WT (+/+) and *Smurf2^T/T^* (T/T) mice (N=14). Error bars are SD. Student’s *t*-test is used for statistical analysis.

**Fig. S2** Average numbers of short-term hematopoietic stem cells (ST-HSCs; Lin^-^Sca1^+^c-kit^++^CD150^-^Flt3^-^), multi-potent progenitors (MPPs; Lin^-^Sca1^+^c-kit^++^CD150^-^Flt3^+^) and LSK (Lin^-^Sca1^+^c-kit^++^) cells in BM of (A) young (2-month, N=11) mice; (B) old (24-month old, N=6) mice. Error bars are SD and Student’s *t*-test is used for statistical analysis.

**Fig. S3** Complete blood count analysis of peripheral blood in young (2-month, N=8) and old (18 to 20-month, N=6) wild-type (+/+) and *Smurf2^T/T^* (T/T) mice. Hb (hemoglobin), HCT (hematocrit), MCV (mean corpuscular volume), MCH (mean corpuscular hemoglobin), MCHC (mean corpuscular hemoglobin concentration), RDW (red blood cell distribution width). Error bars are SD and Student’s *t*–test is used in pairwise comparison. Only *P*<0.05 are indicated. *:<0.05, **: *P*<0.01, ***: *P*<0.001.

**Fig. S4** Enhanced proliferation in the bone marrow (BM) compartments of Smurf2-deficient mice. (A) Cell cycle analysis of total BM cells in wild-type (+/+) and *Smurf2^T/T^* (T/T) mice (N=3). (B) BrdU incorporation in LSK (Lin^-^Sca1^+^c-kit^++^) and progenitor (Lin^-^Sca1^+^c-kit^++^CD150^-^) cells in wild-type (+/+) and *Smurf2^T/T^* (T/T) mice (N=3). (C) Cell cycle analysis of LSK (Lin^-^Sca1^+^c-kit^++^) and progenitor (Lin^-^Sca1^+^c-kit^++^CD150^-^) cells in wild-type (+/+) and *Smurf2^T/T^* (T/T) mice (N=3). Error bars are SD and Student’s *t*–test is used in statistical analysis. (D) Controls for Ki-67 staining. BM cells of a wild-type mouse were stained with the lineage cocktail of antibodies and gated on lineage-negative cells. Plots of DAPI vs. Ki-67 staining are shown from left to right: no Ki-67 staining control, Ki-67 isotype staining control and Ki-67 staining positive control.

**Fig. S5** Donor contributions to bone marrow (BM) and complete blood count analysis of peripheral blood in recipient mice during serial transplantation. (A) Donor (CD45.2^+^) contributions to long-term hematopoietic stem cells (LT-HSCs) in BM of recipient mice 2 month post transplantation. Two independent serial transplantation experiments using male (left panel) or female (right panel) donors are shown. (B) Complete blood count analysis of peripheral blood in recipient mice receiving bone marrow from 2-month old wild-type (+/+) and *Smurf2^T/T^* (T/T) mice during serial transplantation. (C) Complete blood count analysis of peripheral blood in recipient mice receiving bone marrow from 24-month old wild-type (+/+) and *Smurf2^T/T^* (T/T) mice during serial transplantation. Error bars are SD.

**Fig. S6** Kaplan-Meier survival curves of recipient mice receiving bone marrow from 2-month old Smurf2-deficient donors in the 5^th^ transplantation cycle.
